# Trial protocol: RadTARGET, a multicenter phase II randomized controlled trial evaluating focal radiotherapy boost with de-intensification of dose to non-suspicious prostate in patients with intermediate- or high-risk prostate cancer

**DOI:** 10.1016/j.ctro.2026.101184

**Published:** 2026-05-15

**Authors:** Anna M. Dornisch, Mariluz Rojo Domingo, Roberta Vezza Alexander, Christopher C. Conlin, Son Do, Rana R. McKay, Vitali Moiseenko, Michael A. Liss, Jasmine Liu, Todd Pawlicki, Samuel Pena, Edmund M. Qiao, Brent S. Rose, Rhea Rupareliya, Ajay P. Sandhu, Jessica Scholey, Steven N. Seyedin, James J. Urbanic, Lee-Jen Wei, Tyler M. Seibert

**Affiliations:** a Department of Radiation Medicine and Applied Sciences, University of California San Diego, La Jolla, USA; b Moores Cancer Center, University of California San Diego, La Jolla, USA; c Department of Bioengineering, University of California San Diego, La Jolla, USA; d Department of Radiology, University of California San Diego, La Jolla, USA; e Department of Urology, University of California San Diego, La Jolla, USA; f Department of Radiation Oncology, University of California San Francisco, San Francisco, USA; g Department of Biostatistics, Harvard T.H. Chan School of Public Health, Boston, USA

**Keywords:** Pelvic radiation therapy, Focal therapy, Tumor boost, Dose de-escalation, Quality of life, Genitourinary toxicity, Gastrointestinal toxicity, Diffusion MRI, Restriction spectrum imaging, PSMA PET

## Abstract

Definitive radiotherapy (RT) for prostate cancer (PC) with dose intensification and/or focal boosting has excellent oncologic outcomes, but many patients experience adverse events. Dose escalation to the whole prostate improves outcomes at the expense of increased late adverse events. Intraprostatic recurrence after definitive RT typically occurs at the site of the primary tumor, suggesting that dose to the site of the dominant lesion is an important predictor of future failure. The efficacy and safety of tumor-focused RT compared to that of standard RT for definitive treatment of localized PC has not been assessed. RadTARGET (RAdiation Dose TAiloRing Guided by Enhanced Targeting; NCT06990542) is a phase II randomized trial that aims to demonstrate superior safety of image-guided, tumor-focused RT compared to standard RT for acute genitourinary (GU) or gastrointestinal (GI) in the setting of definitive RT for intermediate- and high-risk PC. The study intervention is image-guided, tumor-focused RT with dose intensification of cancer visible on imaging and dose de-intensification to remaining prostate. Patients will be randomized to two arms: those who receive standard RT dose and those that receive tumor-focused RT. The study population will be patients with intermediate- or high-risk PC planning to undergo definitive RT with or without systemic therapy. The primary endpoint to compare between randomized arms is acute GU or GI grade ≥2 adverse events. Participant and study duration are 5 years and 8 years, respectively. RadTARGET will compare the efficacy and safety of tumor-focused RT to that of standard RT for definitive treatment of localized PC. We hypothesize that the tumor-focused approach will substantially reduce adverse events after prostate RT while retaining high efficacy. If this hypothesis is confirmed, we will conclude that a phase III randomized control trial is warranted to formally establish oncologic non-inferiority compared to the current standard of whole-gland dose escalation.

## Highlights

• Phase II trial compares tumor-focused vs standard RT in prostate cancer.
• Image-guided RT boosts tumors and reduces dose outside of tumors.
• Test if image-guided RT reduces acute GU/GI toxicity vs standard RT.
• Target dominant lesion where most post-RT recurrences occur.
• Results will inform phase III trial for efficacy and safety validation.

## Introduction

Modern prostate radiation therapy (RT), with dose-escalation and focal boosting, has excellent oncologic outcomes for patients with intermediate- and high-risk prostate cancer (PC) [1], [2]. Dose escalation to the whole prostate comes at the expense of meaningful adverse events [3], [4], spurring efforts to advance RT delivery to maximize both disease control and safety. The landmark FLAME randomized trial provided high-level evidence that the addition of a focal boost to the MRI-defined tumor improved oncologic outcomes without increased adverse events or decreased quality of life, compared to standard dose to the whole prostate [1]. However, many patients undergoing standard dose to the whole prostate still experience short- and long-term adverse events that impact post-treatment quality of life. Given that patients typically live many years after definitive treatment for localized PC, there is interest in improving the therapeutic ratio of definitive RT for localized PC to minimize impact of treatment on long-term quality of life.

One way to improve the therapeutic ratio is by decreasing rates of adverse events. Side effects of RT are driven by radiation dose to neighboring organs of interest (NOIs; historically called organs at risk), particularly rectum and bladder for definitive prostate radiotherapy [5]. The urethra and penile bulb are also thought to be important [6], [7], [8]. While focal boosting maintains a high probability of tumor control, treatment-related side effects could potentially be reduced through tumor-focused RT. Patterns of failure analyses in the context of both focal boosting and non-focal boosting demonstrate that intra-prostate recurrences occur primarily at the location of the dominant tumor [9], [10], [11], [12]. Furthermore, the FLAME investigators demonstrated a clear dose–effect relationship: near-minimum dose to gross tumor volume (GTV) was associated with risk of PC recurrence [12], [13]. Thus, intra-prostatic recurrence is likely a consequence of undertreatment of the primary tumor rather than undertreatment of the remaining prostate gland, providing a premise for a new approach to focal boosting with tumor-focused RT whereby the tumor receives ablative-dose RT, and the rest of the electively irradiated prostate receives a de-escalated dose.

Accurate discrimination of clinically significant intra-prostate tumors from benign prostate tissue is a prerequisite for tumor-focused RT. Multiparametric magnetic resonance imaging (mpMRI) is the current standard for PC detection [14] and tumor delineation [15]. A quantitative imaging biomarker for PC – the Restriction Spectrum Imaging restriction score (RSIrs) – has been shown to be more specific than conventional mpMRI for clinically significant PC detection [16], [17], [18], [19]. Beyond MRI, positron emission tomography targeting the prostate-specific membrane antigen (PSMA PET) is now routinely used in staging of primary PC [20], and several studies report excellent performance for identification of intra-prostate tumors [21], [22], [23]. Furthermore, the combination of MRI and PSMA PET improve the sensitivity and specificity of intra-prostatic tumor delineation over each modality on its own [24], [25]. By utilizing these advanced biological imaging techniques, intra-prostatic tumors can be accurately delineated with confidence, allowing for tumor-focused RT.

Tumor-focused RT involves ablative-dose RT to the imaging-defined tumors, while de-escalating dose to the remainder of the electively irradiated prostate gland. Similar approaches are commonly used in definitive RT for non-prostate malignancies, such as head and neck cancers. Lack of discrimination on CT scans between normal prostate tissue and PC previously made tumor-focused RT infeasible for PC. However, with improvement in imaging allowing for discrimination of PC versus benign prostate tissue, tumor-focused RT could now be adopted to lower dose to NOIs and potentially increase safety and quality of life while preserving the excellent oncologic outcomes seen with current RT. Prospective data on this topic are scarce, and no randomized trials have been completed. There is one ongoing trial of MRI-guided RT dose de-escalation (DESTINATION) using a linear accelerator with onboard MRI (MR-linac) [26]. A pragmatic, randomized trial with standard linear accelerators is warranted to compare the safety and efficacy of tumor-focused RT to standard RT. We describe here the RAdiotherapy Dose TAiloRing Guided by Enhanced Targeting (RadTARGET) study, a multicenter phase II randomized controlled trial evaluating a tumor-focused strategy for PC radiotherapy (ClinicalTrials.gov NCT06990542). We hypothesize that tumor-focused RT will yield similar efficacy and substantially lower adverse events compared to standard prostate RT.

## Design

**Fig. 1:**
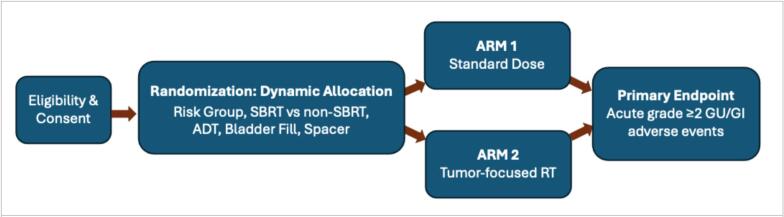
RadTARGET trial schema.

RadTARGET is a phase II, two-arm, randomized clinical trial evaluating tumor-focused RT versus standard-dose RT. Evaluation will be performed in terms of physician-reported adverse events, patient-reported safety, and oncologic outcomes. Participants will undergo RT for intermediate- or high-risk localized PC (Fig. 1). A dynamic allocation procedure is used to assign participants in a 1:1 ratio to receive either standard-dose RT or image-guided, tumor-focused RT. To minimize biased clinical decision-making based on randomization, treating physicians will declare the participant’s treatment plan prior to randomization. Each arm will be balanced for risk group (intermediate/high), fractionation type (stereotactic body RT [SBRT] / non-SBRT), duration of androgen deprivation therapy (ADT; none/short-term/long-term), bladder filling (full/empty), and spacer use (yes/no). The primary objective is to evaluate the relative merits of image-guided, tumor-focused RT vs. standard-dose RT for acute GU/GI adverse events in the setting of definitive RT for intermediate- and high-risk PC. The endpoint will be acute, any-attribution GU/GI grade ≥2 adverse events within 3 months of completing RT, compared between randomized arms. Adverse events will be defined using Common Terminology Criteria for Adverse Events (CTCAE) version 5.0 grading.

The study will enroll 150 participants at the University of California San Diego (UCSD), with completion of enrollment anticipated over 3 years. Inclusion and exclusion criteria are listed in Table 1. Participants must be ≥18 years old, have a diagnosis of intermediate- or high-risk localized PC, and plan to undergo curative-intent RT. They must also have one or more lesions visible on prostate MRI and/or PSMA PET with concordant pathology from biopsy needle locations. Participants with bilateral hip implants, prior PC treatment (including prostatectomy), or prior RT to the pelvis are not eligible. ADT may be started up to 90 days before randomization if study-compatible imaging was performed within 4 months of starting ADT. Participants must provide informed consent and be willing to comply with all study procedures for the duration of the study. This study was approved by UCSD’s Institutional Review Board (IRB) and ethics committee.

**Table 1:** Inclusion and exclusion criteria for the RadTarget trial. The study population is patients with intermediate- or high-risk prostate cancer planning to undergo definitive radiotherapy with or without systemic therapy.

RadTarget Trial Study Population
**Inclusion Criteria**
To be eligible to participate in this study, an individual must meet all of the following criteria:
• Provision of signed and dated informed consent form
• Stated willingness to comply with all study procedures and availability for the duration of the study
• Persons, aged at least 18 years
• Histologic confirmation of prostate adenocarcinoma with plan for curative-intent radiation therapy
• Lesion visible on prostate MRI and/or PSMA PET/CT (according to the treating physician) with concordant pathology from biopsy needle locations
• For participants able to cause a pregnancy: use of condoms or other methods to ensure effective contraception with partner during the radiation therapy treatment and for at least 6 months afterward.

**Exclusion Criteria**
An individual who meets any of the following criteria will be excluded from participation in this study:
• Bilateral hip implants
• Prior prostatectomy
• Prior prostate cancer treatment (e.g., focal therapy). Note that participants who started hormone therapy within the 90 days prior to randomization are eligible, as long as study-compatible imaging was performed within the 4 months prior to starting the hormone therapy.
• Prior radiation therapy to an area requiring treatment in the present study

### Treatment description

The study intervention is image-guided, ablative-dose RT to the sites of clinically significant, macroscopic tumors, while simultaneously de-escalating dose to areas of subclinical disease or benign prostate tissue within the rest of the electively irradiated prostate. All participants must have a pre-treatment MRI (consistent with PI-RADS v2.1 criteria [14]) with a multi-*b*-value diffusion-weighted MRI protocol compatible with calculation of the Restriction Spectrum Imaging restriction score (RSIrs) cancer biomarker. In RadTARGET, the treatment target (gross tumor volume [GTV]) will be manually delineated by the treating radiation oncologist using MRI with RSIrs +/- PSMA PET. Biopsy results and radiologist annotations that may be available should also be considered. For patients receiving ADT, the GTV should be delineated using pre-ADT images. Data collection and follow-up details are provided in the Supplemental Material and Supplemental Table 1.

### Experimental arm

For the experimental arm, clinical target volumes (CTV) are: *CTV_high*, defined as the GTV + 5 mm isotropic margin, cropped within prostate (with optional delineation of additional areas of moderate suspicion); and *CTV_low*, defined as the prostate +/- seminal vesicles (at the discretion of the treating physician). Elective pelvic lymph node RT (*CTV_LN*) is optional and at the discretion of the treating physician. Creation of planning target volumes (PTV) should follow UC San Diego Department of Radiation Medicine and Applied Sciences (RMAS) standards and guidelines, but adaptation is allowed at physician discretion or per local institutional standard practice. *PTV_high* is defined as *CTV_high* without additional margin. Doses for the various fractionations for the experimental arm are listed in Table 2. The dose covering ≥98% of the target volume (D98) for *CTV_high* for participants in the experimental arm must be at least standard of care prescription dose.

**Table 2:** Radiation doses for the experimental arm. Abbreviations: GTV, gross tumor volume (the visible tumor); CTV, clinical target volume (GTV with isotropic margin to cover areas at risk of microscopic disease); Gy, gray.

Fractionation	GTV	CTV_high	CTV_low	CTV_LN
Standard Fractionation (39–40 fx)	D98 up to 94 Gy	78–80 Gy in 39–40 fx	56 Gy in 28 fx	50.4 Gy in 28 fx
Moderate Hypofractionation (28 fx)	D98 up to 84 Gy	70 Gy	56 Gy	50.4 Gy
Moderate Hypofractionation (20 fx)	D98 up to 74 Gy	60 Gy	56 Gy	44 Gy
Stereotactic Body Radiotherapy (5 fx)	D98 up to 45 Gy	40 Gy	30 Gy	25 Gy

### Control arm

As a pragmatic trial investigating image-guided, tumor-focused RT for intermediate- and high-risk prostate cancer, RadTARGET is not overly prescriptive for the control arm. Control arm definitive RT modality, dose, frequency, and administration must be in concordance with RMAS Standards and Guidelines or local institutional standard practice. Image-guided focal boost to MRI-visible disease is allowed and generally expected, but not required. For the control arm, the prostate CTV includes the entire prostate and receives the full prescription dose, corresponding to the relevant allowed fractionation scheme (i.e., *CTV_high* in Table 2). The proximal seminal vesicles may be included at physician discretion and would receive the *CTV_low* dose in Table 2.

### Both arms

For both trial arms, all other CTV and PTV structures should conform to RMAS or local institutional standards and guidelines. NOIs should be delineated per RMAS guidelines or local institutional practice. The urethra must be delineated if a focal tumor boost is delivered. As a pragmatic trial, all plans will be optimized to reflect NOIs constraints of RMAS or local institutional standards & guidelines. RMAS guidelines NOIs constraints for all fractionation schemes are provided in the Supplement. The treating physician must state their intended radiation technique, number of fractions, coverage of lymph nodes, use of adaptive RT, and plan for spacer prior to randomization. Systemic therapy (e.g., ADT) is allowed at discretion of the treating physician, who must state the intended medications and duration prior to randomization (see Supplement).

### Radiation therapy simulation (both arms)

Simulation allows for computer-based optimization of radiation dose delivery to the target tissue and simultaneous dose minimization to surrounding normal tissues. In both arms, simulation with CT and/or MRI is performed by RT-trained MR technologists per clinical routine. The diagnostic MRI +/- PSMA PET/CT are registered to the CT simulation for target delineation. A post-ADT planning MRI close to the date of the simulation is required for patients who began ADT more than 3 weeks prior to simulation. Full or empty bladder protocols are acceptable.

### Radiation therapy planning and delivery (both arms)

**Fig. 2:**
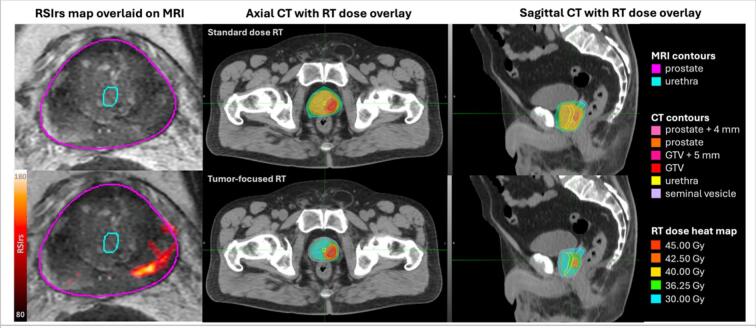
Comparison of standard dose and tumor-focused radiotherapy (RT) plans on computed tomography (CT) images for the RadTARGET trial. Magnetic resonance images (MRI) are used to localize the tumor for intensified treatment dose at the GTV (and for prostate and urethra contours). Restriction spectrum imaging restriction score (RSIrs) maps (overlaid on T2-weighted images) help radiation oncologists target the tumor. In the control arm, standard RT dose is applied to the entire prostate (with a uniform margin of 4 mm). Image-guided focal boost to MRI-visible disease is allowed and generally expected. In the experimental arm, the dose is increased at the tumor (with a uniform margin of 5 mm, limited to within the prostate) and decreased outside of the tumor. The patient shown was treated with Stereotactic Body Radiation Therapy (SBRT). Abbreviations: GTV = gross tumor volume, Gy = gray.

RT planning is performed by trained dosimetrists and/or radiation physicists (complying with RMAS guidelines or local institutional practice) and documented in the medical records. Peer review of RT plans by radiation physicists and at least one radiation oncologist is required, as well as contouring of all NOIs within 5 cm of any target volume. All plans must use modern planning and delivery techniques (e.g., VMAT), regardless of fractionation (conventional, hypofractionated, or SBRT). Target dose and/or coverage should be lowered to meet hard NOI dose goals, as needed (dose goals listed in Supplemental Tables 2-4). NOI dose goals may be exceeded in the patient’s interests at discretion of the treating physician. RT delivery is performed by trained RT therapists. A comparison of standard and tumor-focused plans for an example patient is shown in Fig. 2.

### Study endpoints

• Primary Endpoint: physician-reported, all-attribution acute GI or GU grade ≥2 adverse events. Adverse events will be defined using CTCAE version 5.0 grading. This study will evaluate the relative merits of standard dose RT and image-guided tumor-focused RT in terms of safety, patient-reported outcomes, and oncologic efficacy.
• Secondary Endpoints:
o Acute radiation-attribution GU/GI grade ≥2 adverse events.
o Acute radiation-attribution grade ≥2 GU adverse events, other than alpha blockers.
o Acute any-attribution and radiation-attribution grade ≥3 GU/GI adverse events.
o Late any attribution and radiation-attribution grade ≥2 and grade ≥3 GU/GI adverse events.
o Patient-reported urinary and bowel quality of life, defined as a decrease of 7 points and 6 points on the irritative/obstructive and bowel domains, respectively, of EPIC-26 at 3 months, 6 months, 12, 18, and 24 months after completion of RT.
o Biochemical recurrence free survival at 5 years, defined as the percentage of participants alive and without biochemical at 5 years after completion of RT.
o Local failure at 5 years, defined as the percentage of participants with intra-prostatic recurrence (as assessed by MRI and/or PSMA PET/CT or via confirmatory biopsy after biochemical recurrence) at 5 years after completion of RT.
o Distant-metastasis-free survival at 5 years, defined as absence of local or regional lymph node recurrence (as confirmed on imaging including PSMA PET/CT and mpMRI) at 5 years after completion of RT.
o Overall survival at 5 years, defined as the percentage of patients alive at 5 years after completion of RT.

### Statistical analysis

We adopt a statistical approach based on estimating the difference in adverse event rate (proportion of patients experiencing an adverse event) between the two randomized groups by controlling the precision (width) of the associated confidence interval for that difference. The primary endpoint is acute GU or GI grade ≥2 adverse events. We will estimate the absolute difference in adverse event rate between tumor-focused radiotherapy and standard dose radiotherapy using an intention-to-treat (ITT) analysis approach.

The following randomized trials and meta-analyses guide our assumptions for sample size determinations: RTOG 0415, PROFIT, HYPRO, PACE-B, FLAME, Hypo-FLAME, and MIRAGE [2], [4], [13], [27], [28], [29], [30]. From this high-level evidence, we assume the combined GU and/or GI acute adverse event rate is 40%. The MIRAGE trial included 156 participants and found a meaningful difference in acute GU and GI safety when comparing a difference in PTV margin size of 2 mm. We assume that the tumor-focused approach will lead to a similar adverse event reduction via the same mechanism.

We seek to answer whether the tumor-targeted approach reduces the rate of acute GU/GI adverse events. If we assume acute adverse events are reduced from 40% to 25% (absolute rate difference of −15%), we estimate that a sample size of 150 participants (75 in each arm) will yield a one-sided 90% confidence interval (one-sided equivalent to 95% confidence interval for a two-sided test) with 12.4% margin of error. Thus, we expect to have >95% confidence that the true rate difference is non-zero. We expect minimal attrition, but even 10% missing data would have a minimal impact on the margin of error (<1%). If adverse event rates in the standard arm were higher or lower than expected (30–70%), we would still have sufficient power to detect the assumed 15% absolute rate difference.

All primary and secondary analyses will be conducted with the intention-to-treat principle. All participants will be analyzed according to the group they were randomly assigned to, regardless of compliance with the trial protocol. Patients who did not receive RT treatment will be considered screen failures. The primary endpoints (proportion of patients with acute grade ≥2 GU or GI adverse events) will be compared across the two randomized groups using a stratified Cochran–Mantel–Haenszel test with the stratification factors listed above. We will also repeat the analysis using a non-stratified Fisher’s exact test.

Secondarily, we will develop a multivariable logistic regression model with binary dependent variable (presence or absence of acute grade ≥2 GU or GI adverse events) and independent variables to include randomization group and all covariates listed above. This will allow us to estimate the effects of each variable, with bootstrap 95% confidence intervals. The primary and secondary analyses above will be repeated in the following subgroups: (1) self-reported race/ethnicity, and (2) age <65 vs. ≥65 years. This is in addition to subgroup analyses within the stratification groupings for randomization. Description of translational sub-studies provided in the Supplement.

## Summary

RadTARGET is a phase II randomized trial designed to evaluate the safety and efficacy of a novel, tumor-focused approach to definitive RT for localized PC. The study leverages recent advances in imaging and RT delivery to individualize treatment plans for each patient with a goal of maintaining excellent efficacy while reducing adverse events compared to standard RT. Importantly, we designed this trial with an eye toward improving treatment for most patients undergoing definitive RT for PC, not limited to patients with access to disease-site subspecialists at tertiary referral academic centers or with specialized, expensive equipment, such as an MR-Linac. Standard linear accelerators are used in RadTARGET. The FLAME patterns of recurrence analysis found that intra-prostatic recurrences overlapped with the GTV visible on pre-treatment MRI, underscoring that accurate delineation of MRI-visible tumors is critical for treatment success [13]. However, significant inter-observer variability in tumor contouring remains a challenge in clinical practice, especially when relying solely on conventional mpMRI [15]. While *important* in focal boosting, standardized, reproducible, and accurate tumor contours are *imperative* in tumor-focused RT to ensure consistency in treatment delivery and preservation of excellent oncologic outcomes.

For the GTV delineation in this trial, we mandate use of an advanced MRI biomarker (RSIrs), which provides superior tumor conspicuity, has been validated against histopathology in multiple studies, and is quantitative, allowing for standardized, reproducible thresholds in tumor identification [16], [17], [18], [19]. While RSIrs is not widely available at present, it is a potentially scalable solution that can be implemented on modern clinical MRI scanners from any vendor. Thus, not only is RadTARGET one of the first randomized trials evaluating tumor-focused RT, but it is also the first randomized trial using a quantitative MRI biomarker to guide radiation target volumes in PC. Beyond MRI, our trial also allows PSMA PET/CT, if available, to complement MRI for GTV delineation, as the combination of MRI and PSMA PET improved the sensitivity and specificity of intra-prostatic tumor delineation over each modality on its own [24], [25]. Our contouring strategy may be updated in the future if additional high-level data justify adjustments.

In conclusion, RadTARGET is a multi-center phase II randomized controlled trial evaluating an innovative, tumor-focused RT strategy for patients with intermediate- or high-risk PC. We hypothesize that the tumor-focused approach will substantially reduce adverse events after prostate RT while retaining high efficacy. If this hypothesis is confirmed, we will conclude that a phase III randomized controlled trial is warranted to formally establish oncologic non-inferiority compared to the current standard of whole-gland dose escalation.

## CRediT authorship contribution statement

**Anna M. Dornisch:** Conceptualization, Data curation, Formal analysis, Investigation, Methodology, Resources, Software, Visualization, Writing – original draft, Writing – review & editing. **Mariluz Rojo Domingo:** Conceptualization, Data curation, Formal analysis, Investigation, Methodology, Resources, Software, Validation, Visualization, Writing – original draft, Writing – review & editing. **Roberta Vezza Alexander:** Data curation, Resources, Software, Visualization, Writing – review & editing. **Christopher C. Conlin:** Data curation, Resources, Software, Visualization, Writing – review & editing. **Son Do:** Data curation, Resources, Software, Visualization, Writing – review & editing. **Rana R. McKay:** Data curation, Resources, Software, Visualization, Writing – review & editing. **Vitali Moiseenko:** Data curation, Resources, Software, Visualization, Writing – review & editing. **Michael A. Liss:** Data curation, Resources, Software, Visualization, Writing – review & editing. **Jasmine Liu:** Data curation, Resources, Software, Visualization, Writing – review & editing. **Todd Pawlicki:** Data curation, Resources, Software, Visualization, Writing – review & editing. **Samuel Peña:** Data curation, Resources, Software, Visualization, Writing – review & editing. **Edmund M. Qiao:** Data curation, Resources, Software, Visualization, Writing – review & editing. **Brent S. Rose:** Data curation, Resources, Software, Visualization, Writing – review & editing. **Rhea Rupareliya:** Data curation, Resources, Software, Visualization, Writing – review & editing. **Ajay P. Sandhu:** Data curation, Resources, Software, Visualization, Writing – review & editing. **Jessica Scholey:** Data curation, Resources, Software, Visualization, Writing – review & editing. **Steven N. Seyedin:** Data curation, Resources, Software, Visualization, Writing – review & editing. **James J. Urbanic:** Data curation, Resources, Software, Visualization, Writing – review & editing, Writing – review & editing. **Lee-Jen Wei:** Data curation, Resources, Software, Visualization, Writing – review & editing. **Tyler M. Seibert:** Conceptualization, Data curation, Formal analysis, Funding acquisition, Investigation, Methodology, Project administration, Resources, Software, Supervision, Validation, Visualization, Writing – original draft, Writing – review & editing.

## Funding

This work was funded by UC San Diego School of Medicine and UC San Diego Moores Cancer Center.

## Declaration of competing interest

The authors declare the following financial interests/personal relationships which may be considered as potential competing interests: Dr. Tyler Seibert reports honoraria or consulting fees from Varian Medical Systems, WebMD, MJH Life Sciences, MD Education USA, GE Healthcare, Blue Earth Diagnostics, Janssen, CorTechs Labs, and MyOme. He has stock options in CorTechs Labs, MyOme, and Open Medicine for serving on their scientific advisory boards. He receives research funding and/or in-kind research support from GE Healthcare, Blue Earth Diagnostics, Quibim, AIRA Matrix, Veracyte, and Lantheus, all through the University of California San Diego. These companies might potentially benefit from the research results. The terms of this arrangement have been reviewed and approved by the University of California San Diego in accordance with its conflict-of-interest policies. Dr. Rana McKay serves on the advisory boards of Janssen, Novartis, Tempus, Pfizer, Astellas Medivation, Dendreon, Bayer, Sanofi, Vividion Therapeutics, Calithera Biosciences, Caris Life Sciences, Sorrent Therapeutics, AVEO, Seagen, Telix Pharmaceuticals, Lilly, Blue Earth Diagnostics, Ambrx, Sumitomo Pharma Oncology, Eisai, NeoMorph, Arcus Biosciences, Daiichi Sankyo, Exelixis, Bristol-Myers Squibb, AstraZeneca, Merck, Myovant Sciences and Precede Bio. She also receives research funding from Bayer, Tempus, AstraZeneca, Exelixis, Bristol-Meyers Squibb, Oncternal Therapeutics and Artera. Dr. Michael Liss is the founder of Oncobiomix and receives research funding from MicrogenDx.
