# Supplementary material for: Trial protocol: RadTARGET, a multicenter phase II randomized controlled trial evaluating focal radiotherapy boost with de-intensification of dose to non-suspicious prostate in patients with intermediate- or high-risk prostate cancer
Source: Clin Transl Radiat Oncol. 2026 May 15;59:101184. doi: 10.1016/j.ctro.2026.101184 (PMC13202547; doi:10.1016/j.ctro.2026.101184)
Supplement: Supplementary Data 1 — Additional protocol details are provided in the Supplementary Material. [file mmc1.docx]

**Supplementary Material**

**DESIGN**

Systemic Therapy

Systemic therapy (e.g., ADT) use and duration are left to the discretion of the treating physician in this pragmatic trial. The intended use of ADT and/or other systemic therapy is stated by the treating physician prior to randomization. In case of administration of ADT, the GTV should be delineated under consideration of pre-ADT images. For patients who began ADT ≥3 weeks prior to simulation, a post-ADT planning MRI is required to be done reasonably close to the date of the simulation (preferably within 1 week). For analysis, planned ADT is classified into three groups: 1) no ADT, 2) short-term ADT (9 months or less), and long-term ADT (9 months or more). In case of administration of ADT, the GTV should be delineated under consideration of pre-ADT images. For patients who began ADT ≥3 weeks prior to simulation, a post-ADT planning MRI is required to be done reasonably close to the date of the simulation (preferably within 1 week).

Patients must also be on a stable dose of any medications to treat urinary symptoms, such as alpha-blockers of anticholinergic drugs, for at least 14 days prior to completion of baseline patient-reported outcomes questionnaires. After initiation of study protocol, medication to treat new urinary symptoms may be prescribed at discretion of treating physician (and will be recorded).

Data Collection and Follow-Up: Pre-therapeutic work-up and screening assessment

Screening evaluations must be performed within 42 days prior to randomization. Inclusion and exclusion criteria are checked and validated. The complete screening work-up includes medical history, medication review, demographic information, vital signs, performance status, and standard laboratory tests. Patients will report baseline quality of life assessment using EPIC-26, FACT-G, and prostate FACT questionnaires. When baseline assessments are completed, validation of inclusion and exclusion criteria for the study is performed followed by stratification parameter assessment. Patients are then randomized into tumor-focused RT or standard therapy arms.

Data Collection and Follow-Up: Assessments during treatment phase

Patients undergo clinical assessments while undergoing RT per standard care clinical practice. On the last day of radiation treatment, patients undergo a study assessment. This will comprise of a medication review, vital signs, performance status, a provider adverse events assessment using the CTCAE v5.0 criteria, and a patient reported quality of life assessment using EPIC-26, FACT-G, and prostate-specific FACT (if applicable) questionnaires.

Data Collection and Follow-Up: Assessments during the follow-up phase

Patients will have follow-up visits at 3, 6*,* 12, 18*,* and 24 months after randomization. At each follow-up visit, providers will assess medical history, medication review, performance status, and adverse events using CTCAE v5.0. This can be done via telehealth or in person. Additionally, patients undergo PSA +/- testosterone testing. Lastly, patients complete quality of life assessments using EPIC-26, FACT-G, and prostate-specific FACT questionnaires. Afterwards, long-term follow-up is conducted every 6 months (± 14 days) for at least 5 years after completion of RT to collect data on recurrence, metastasis, and survival. Long-term follow-up data can be extracted from the medical records. During the entire study, biochemical cancer recurrence is assessed by testing for PSA +/- testosterone level (testosterone measurement is optional during the long-term follow-up). Assessment of tumor recurrence and metastatic disease with MRI and/or PSMA PET or confirmatory biopsy after biochemical recurrence is done at the discretion of the treating physician.

Data Collection and Follow-Up: Assessment of oncologic outcomes

Oncologic outcomes are assessed via PSA monitoring followed by routine clinical imaging and/or biopsy, if deemed appropriate at the discretion of the treating physician team. Biochemical failure is defined according to the Phoenix definition, and biochemical recurrence-free survival will be assessed. Rates of local failure will be assessed, and local failure will be defined as rate of intra-prostatic recurrence (as assessed on MRI and/or PSMA PET/CT or via confirmatory biopsy after biochemical recurrence). We will consider a local failure a primary site recurrence using the definition from the patterns of intra-prostatic failure analysis from FLAME with any overlap between the macroscopic recurrent tumor and the original *CTV_high*. A local failure will be a marginal miss if there is abutment between the macroscopic recurrent tumor and the original *CTV_high* without overlap. We will also assess distant metastasis-free survival (MFS) and regional + distant MFS (per FLAME secondary analysis), as confirmed on imaging, preferably PSMA PET/CT or mpMRI. Overall survival will also be assessed. Assessments will occur as per the Schedule of Activities described in Supplementary Table 1.

**STATISTICAL ANALYSIS**

Translational Sub-studies

There is increasing interest in finding biomarkers predictive of cancer-directed therapy adverse events, including chemotherapy^1^ and RT^2^. Recently, radiogenomics studies have been identifying genetic markers that are associated with probably of treatment-related adverse events^3–6^. However, a recent systematic review found a large proportion of studies on correlation of genetic markers with radiotherapy-related side effects did not consider any dosimetric parameters^7^. There is a need for radiogenomics studies with complete information of dosimetric parameters as adverse events have been shown to be dose-dependent^8–10^. The focus of our biomarker investigation will include genomic analysis of peripheral blood samples for germline DNA, ctDNA, host immune cells, and tumor DNA. This allows for less invasive liquid biopsies that offer practical benefits including ease of collection and ability to measure tumor characteristics at multiple time points.

A genomic biomarker based on tumor expression of 22 genes (Decipher by Veracyte, South San Francisco, USA) is being evaluated as mechanism for treatment intensification/de-intensification in cooperative group trials for intermediate and high-risk prostate cancer. We will evaluate the Decipher score and its associated Decipher Genomics Resource for Intelligent Discovery (GRID) platform. There could be higher concern about the tumor-focused approach in patients with high-Decipher tumors. We will conduct subgroup analyses for those with low/intermediate (<0.60) vs. high (≥0.60) Decipher to learn whether a tumor-focused approach is more appropriate for tumors with lower (or higher) Decipher score. PORTOS is another score derived from the Decipher assay, with range -1 to +1, where higher scores have been shown to be associated with improved outcomes with dose-escalated radiotherapy^11^. However, higher PORTOS scores are also associated with worse genitourinary adverse events^12^. We hypothesize that those with higher PORTOS score (>0), indicating greater radiation response will favor a more focused approach and will have better outcomes in terms of reduced genitourinary adverse events.

Beyond liquid biopsies and tumor tissue, previous studies have reported that the microbiome may affect efficacy and safety of cancer therapy. A group of patient microbiomes was identified that may have a higher risk of gastrointestinal side effects during radiation therapy^13^. We invite trial participants to optionally provide samples for microbiome analysis and interested participants consent to sample collection.

**Supplemental Tables**

| 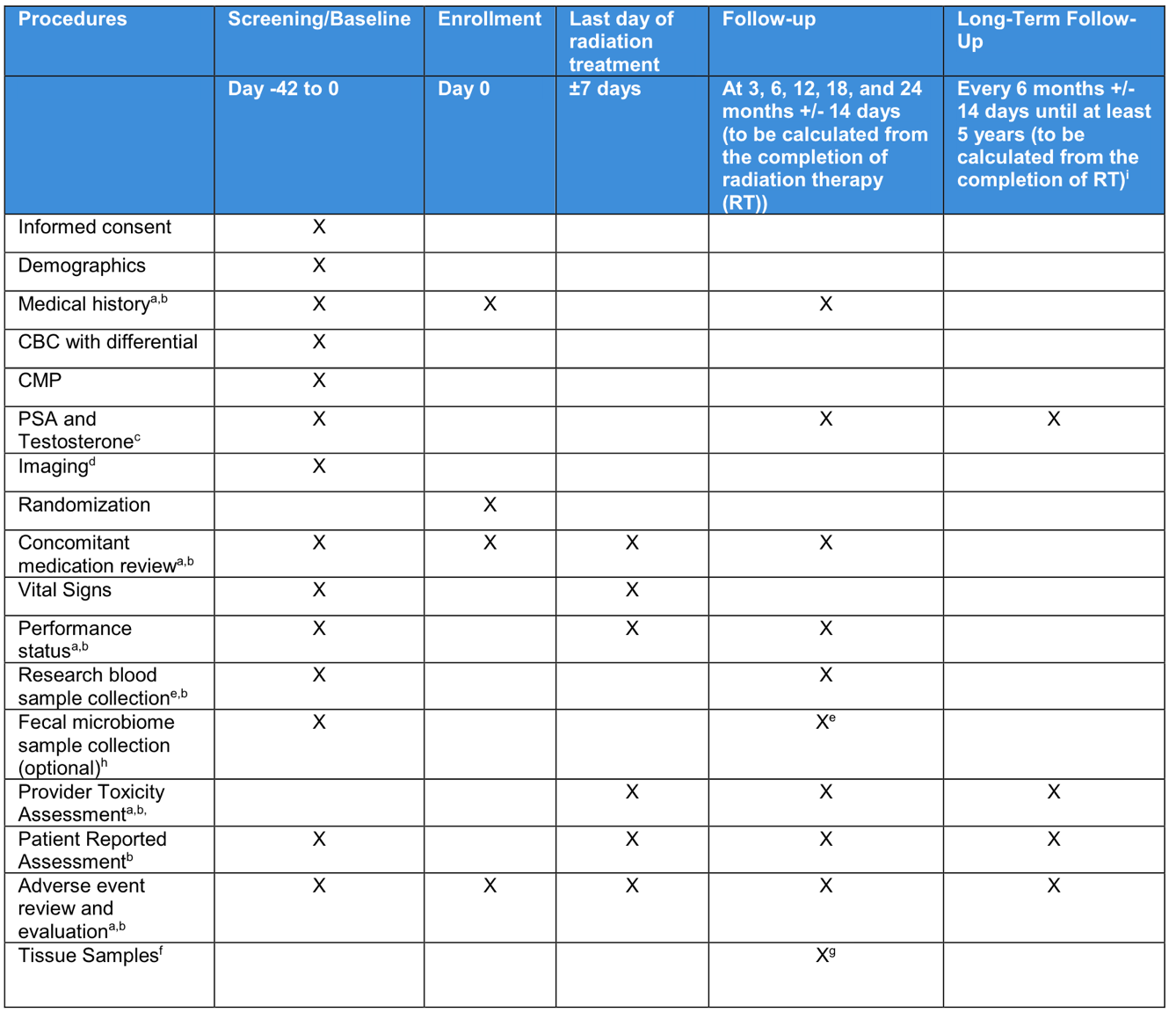 |
| --- |
| **Supplementary Table 1**. Schedule of activities. a: Via provider assessment, either telehealth or in person. b: After 12 months from completion of radiotherapy, it may be performed annually instead of every 6 months at investigator discretion. c: Testosterone measurement is optional for patients who do not undergo androgen deprivation therapy. Testosterone is also optional for all participants in long-term follow-up (i.e., >24 months after completing RT). d: Study-compatible MRI and (optional) PSMA PET/CT can be done within the 7 months prior to randomization. Hormone therapy may have begun within the 90 days prior to randomization, as long as study-compatible MRI and PSMA PET/CT were performed within the 4 months prior to starting the hormone therapy. It is preferred that pre-treatment imaging be performed within the 3 months prior to starting treatment. If hormone therapy precedes RT by ≥3 weeks, a new MRI must be obtained within 3 weeks of initiation of RT (preferably within 1 week). e: Research blood sample collection and optional stool sample collection will be performed at baseline and at 3-, 12-, and 24-months follow-up at time of standard of care lab draw. Baseline research blood samples can be collected any time before starting RT. f: From area of highest tumor content from prior biopsy or surgery: (a) 3 stained H&E slides and (b) 1 block or 30 unstained slides. g: Collection of archived tissue samples will be performed at an unscheduled visit. h: We invite study participants to optionally provide stool samples for exploratory microbiome analysis. i: Via chart review, phone call, telemedicine, or other means of secure communication with the participant to assess subsequent therapies and survival status. Continues until at least 5 years after completion of RT. |

| \| **Neighboring Organ of Interest** \| **Dose Goals** \| \| \| **Reference** \| \| --- \| --- \| --- \| --- \| --- \| \| **Parameter** \| **Soft Goal** \| **Firm Goal** \| \| Rectum \| D_1cc_ \|  \| ≤ 82Gy \| FLAME \| \| V_70Gy_ \|  \| ≤ 20% \| QUANTEC \| \| D_0.03cc_ \|  \| ≤ 96Gy [=120%] \| GU009 \| \| V_60Gy_ \| ≤ 25% \|  \| UCSD \| \| V_40Gy_ \| ≤ 35% \|  \| UCSD \| \| Bladder \| D_1cc_ \|  \| ≤ 86Gy \| FLAME adaptation \| \| D_0.03cc_ \|  \| ≤ 96Gy [=120%] \| GU009 \| \| V_70Gy_ \| ≤ 25% \|  \| UCSD \| \| V_60Gy_ \| ≤ 35% \|  \| UCSD \| \| V_40Gy_ \| ≤ 45% \|  \| UCSD \| \| Urethra (Boost Cases) \| D_0.03cc_ \|  \| ≤ 86Gy \| UCSD \| \| D_0.10cc_ \|  \| ≤ 84Gy \| FLAME \| \| Penile Bulb \| D_mean_ \| < 52.5Gy \|  \| GU009 \| \| Femur R/L \| V_50Gy_ \| < 10% \|  \| GU009 \| \| Bowel_Sigmoid \| D_1cc_ \| < 60Gy \|  \| UCSD \| \| D_0.03cc_ \|  \| < 66Gy \| UCSD \| \| Bowel_Space \| D_0.03cc_ \| < 54Gy \|  \| UCSD \| |
| --- | --- | --- | --- | --- | --- | --- | --- | --- | --- | --- | --- | --- | --- | --- | --- | --- | --- | --- | --- | --- | --- | --- | --- | --- | --- | --- | --- | --- | --- | --- | --- | --- | --- | --- | --- | --- | --- | --- | --- | --- | --- | --- | --- | --- | --- | --- | --- | --- | --- | --- | --- | --- | --- | --- | --- | --- | --- | --- | --- | --- | --- | --- | --- | --- | --- | --- | --- | --- | --- | --- | --- | --- | --- | --- | --- | --- | --- | --- | --- | --- | --- | --- | --- |
| **Supplementary Table 2**. Dose goals for neighboring organs of interest. Prostate: Standard Fractionation (1.8 – 2.0 Gy / fraction). |

| \| **Neighboring Organ of Interest** \| **Dose Goals** \| \| \| **Reference** \| \| --- \| --- \| --- \| --- \| --- \| \| **Parameter** \| **Soft Goal** \| **Firm Goal** \| \| Rectum \| D_1cc_ \|  \| ≤ 73Gy \| FLAME \| \| V_70Gy_ \|  \| ≤ 10% \| GU009, GU010 \| \| D_0.03cc_ \|  \| ≤ 84Gy [=120%] \| GU009 \| \| V_60Gy_ \| ≤ 20% \|  \| UCSD \| \| V_40Gy_ \| ≤ 30% \|  \| UCSD \| \| Bladder \| D_1cc_ \|  \| ≤ 76Gy \| FLAME adaptation \| \| D_0.03cc_ \|  \| ≤ 84Gy [=120%] \| GU009 adaptation \| \| V_70Gy_ \| ≤ 15% \|  \| UCSD \| \| V_60Gy_ \| ≤ 25% \|  \| UCSD \| \| V_40Gy_ \| ≤ 35% \|  \| UCSD \| \| Urethra (Boost Cases) \| D_0.03cc_ \|  \| ≤ 76Gy \| UCSD \| \| D_0.10cc_ \|  \| ≤ 74Gy \| FLAME \| \| Penile Bulb \| D_mean_ \| < 51Gy \|  \| UCSD \| \| D_3cc_ \| < 27.86Gy \|  \| UCSD \| \| Femur R/L \| D_10cc_ \| < 27.86Gy \|  \| UCSD \| \| V_45Gy_ \| ≤ 5% \| ≤ 10% \| GU009 \| \| Bowel_Sigmoid \| D_1cc_ \| < 60Gy \|  \| UCSD \| \| D_0.03cc_ \|  \| < 66Gy \| UCSD \| \| Bowel_Space \| D_0.03cc_ \| < 54Gy \|  \| UCSD \| |
| --- | --- | --- | --- | --- | --- | --- | --- | --- | --- | --- | --- | --- | --- | --- | --- | --- | --- | --- | --- | --- | --- | --- | --- | --- | --- | --- | --- | --- | --- | --- | --- | --- | --- | --- | --- | --- | --- | --- | --- | --- | --- | --- | --- | --- | --- | --- | --- | --- | --- | --- | --- | --- | --- | --- | --- | --- | --- | --- | --- | --- | --- | --- | --- | --- | --- | --- | --- | --- | --- | --- | --- | --- | --- | --- | --- | --- | --- | --- | --- | --- | --- | --- | --- | --- | --- | --- | --- | --- | --- | --- | --- |
| **Supplementary Table 3**. Dose goals for neighboring organs of interest. Prostate: Moderate Hypofractionation (2.5 Gy / fraction). |

| \| **Neighboring Organ of Interest** \| **Dose Goals** \| \| \| **Reference** \| \| --- \| --- \| --- \| --- \| --- \| \| **Parameter** \| **Soft Goal** \| **Firm Goal** \| \| Rectum \| V_36Gy_ \|  \| ≤ 1cc \| PACE-B (*much stricter than GU009/GU010) \| \| D_0.03cc_ \| ≤ 40Gy \| ≤ 41.2Gy \| GU010 \| \| D_1cc_ \| ≤ 36Gy \|  \| UCSD \| \| D_3cc_ \| ≤ 34Gy \|  \| UCSD \| \| D_10%_ \| ≤ 33Gy \|  \| UCSD \| \| D_20%_ \| ≤ 29Gy \|  \| UCSD \| \| D_50%_ \| ≤ 18Gy \|  \| UCSD \| \| Bladder \| V_37Gy_ \|  \| ≤ 10cc \| PACE-B (*much stricter than GU009/GU010) \| \| D_0.03cc_ \|  \| ≤ 43.5Gy \| GU010 \| \| D_1cc_ \| ≤ 38Gy \|  \| UCSD \| \| D_10%_ \| ≤ 33Gy \|  \| UCSD \| \| D_50%_ \| ≤ 18Gy \|  \| UCSD \| \| Urethra (Boost Cases) \| D_0.03cc_ \|  \| ≤ 43.5Gy \| GU010 (*much stricter than PACE-B) \| \| D_0.03cc_ \| ≤ 42Gy \|  \| Hypo-FLAME \| \| V_42Gy_ \| ≤ 50% \|  \| PACE-B \| \| Penile Bulb \| D_mean_ \| < 29.5Gy \|  \| PACE-B \| \| Femur R+L \| D_5%_ \| < 14.5 Gy \|  \| UCSD \| \| D_10cc_ \|  \| < 20Gy \| UCSD \| \| D_15%_ \|  \| ≤ 14.5Gy \| UCSD \| \| D_40%_ \| < 5% \|  \| GU009 \| \| Bowel_Sigmoid \| D_1cc_ \|  \| < 30Gy \| PACE-B \| \| D_5cc_ \|  \| < 18.1Gy \| PACE-B \| |
| --- | --- | --- | --- | --- | --- | --- | --- | --- | --- | --- | --- | --- | --- | --- | --- | --- | --- | --- | --- | --- | --- | --- | --- | --- | --- | --- | --- | --- | --- | --- | --- | --- | --- | --- | --- | --- | --- | --- | --- | --- | --- | --- | --- | --- | --- | --- | --- | --- | --- | --- | --- | --- | --- | --- | --- | --- | --- | --- | --- | --- | --- | --- | --- | --- | --- | --- | --- | --- | --- | --- | --- | --- | --- | --- | --- | --- | --- | --- | --- | --- | --- | --- | --- | --- | --- | --- | --- | --- | --- | --- | --- | --- | --- | --- | --- | --- | --- | --- | --- | --- | --- | --- |
| **Supplementary Table 4**. Dose goals for neighboring organs of interest. Prostate: SBRT (5 fractions). |

**References**

1. Boguszewicz Ł. Predictive Biomarkers for Response and Toxicity of Induction Chemotherapy in Head and Neck Cancers. *Front Oncol*. 2022;12:900903. doi:10.3389/fonc.2022.900903

2. Rosenstein BS. Radiogenomics: Identification of Genomic Predictors for Radiation Toxicity. *Semin Radiat Oncol*. 2017;27(4):300-309. doi:10.1016/j.semradonc.2017.04.005

3. Alsner J, Andreassen CN, Overgaard J. Genetic Markers for Prediction of Normal Tissue Toxicity After Radiotherapy. *Seminars in Radiation Oncology*. 2008;18(2):126-135. doi:10.1016/j.semradonc.2007.10.004

4. Cesaretti JA, Stock RG, Lehrer S, et al. *ATM* sequence variants are predictive of adverse radiotherapy response among patients treated for prostate cancer. *International Journal of Radiation Oncology*Biology*Physics*. 2005;61(1):196-202. doi:10.1016/j.ijrobp.2004.09.031

5. Cesaretti JA, Stock RG, Atencio DP, et al. A Genetically Determined Dose–Volume Histogram Predicts for Rectal Bleeding among Patients Treated With Prostate Brachytherapy. *International Journal of Radiation Oncology*Biology*Physics*. 2007;68(5):1410-1416. doi:10.1016/j.ijrobp.2007.02.052

6. Yuan X, Liao Z, Liu Z, et al. Single nucleotide polymorphism at rs1982073:T869C of the TGFbeta 1 gene is associated with the risk of radiation pneumonitis in patients with non-small-cell lung cancer treated with definitive radiotherapy. *J Clin Oncol*. 2009;27(20):3370-3378. doi:10.1200/jco.2008.20.6763

7. Yahya N, Chua XJ, Manan HA, Ismail F. Inclusion of dosimetric data as covariates in toxicity-related radiogenomic studies. *Strahlenther Onkol*. 2018;194(8):780-786. doi:10.1007/s00066-018-1303-5

8. Matsuo Y, Shibuya K, Nakamura M, et al. Dose--volume metrics associated with radiation pneumonitis after stereotactic body radiation therapy for lung cancer. *Int J Radiat Oncol Biol Phys*. 2012;83(4):e545-9. doi:10.1016/j.ijrobp.2012.01.018

9. Yahya N, Ebert MA, Bulsara M, et al. Urinary symptoms following external beam radiotherapy of the prostate: Dose–symptom correlates with multiple-event and event-count models. *Radiotherapy and Oncology*. 2015;117(2):277-282. doi:10.1016/j.radonc.2015.10.003

10. Yoon H, Oh D, Park HC, et al. Predictive factors for gastroduodenal toxicity based on endoscopy following radiotherapy in patients with hepatocellular carcinoma. *Strahlenther Onkol*. 2013;189(7):541-546. doi:10.1007/s00066-013-0343-0

11. Dal Pra A, Ghadjar P, Ryu HM, et al. Predicting dose response to prostate cancer radiotherapy: validation of a radiation signature in the randomized phase III NRG/RTOG 0126 and SAKK 09/10 trials. *Ann Oncol*. 2025;36(5):572-582. doi:10.1016/j.annonc.2025.01.017

12. Hoffman KE, Kamran SC, Ryu HM, et al. PORTOS gene signature as a predictor of risk of adverse events after dose-escalated vs. lower-dose prostate radiation therapy in NRG/RTOG 0126. *JCO*. 2025;43(5_suppl):375-375. doi:10.1200/JCO.2025.43.5_suppl.375

13. The Role of Gut Microbiota in Modulating Cancer Therapy Efficacy - Madkour - 2024 - Advanced Gut & Microbiome Research - Wiley Online Library. Accessed May 15, 2026. https://onlinelibrary.wiley.com/doi/full/10.1155/2024/9919868
